# Closing the gap — development of an analytical methodology using volumetric absorptive microsampling of finger prick blood followed by LC-HRMS/MS for adherence monitoring of antihypertensive drugs

**DOI:** 10.1007/s00216-022-04394-9

**Published:** 2022-11-01

**Authors:** Cathy M. Jacobs, Michael Kunz, Felix Mahfoud, Lea Wagmann, Markus R. Meyer

**Affiliations:** 1 Department of Experimental and Clinical Toxicology, Institute of Experimental and Clinical Pharmacology and Toxicology, Center for Molecular Signaling (PZMS), Saarland University, Homburg, Germany; 2 Klinik für Innere Medizin III (Kardiologie, Angiologie und Internistische Intensivmedizin), Universitätsklinikum des Saarlandes, Saarland University, Homburg, Germany; 3 Institute for Medical Engineering and Science, MIT, Cambridge, MA USA

**Keywords:** Adherence monitoring, Microsampling, Novel VAMS application, Antihypertensive drugs, LC-HRMS/MS, Analytical applicability

## Abstract

Volumetric absorptive microsampling (VAMS), an emerging microsampling technique, is a promising tool for adherence monitoring. This study focused on development of an analytical methodology to improve VAMS-based strategies for adherence assessment by analyzing angiotensin-converting-enzyme (ACE) inhibitors, loop diuretics, a potassium-sparing diuretic, and a thiazide diuretic. Development included sample preparation, chromatographic conditions, mass spectrometry settings, validation, and demonstrating proof of concept. Quantification of analytes, by name furosemide, hydrochlorothiazide, lisinopril, torasemide, and the active metabolites, canrenone, enalaprilat, and ramiprilat in finger prick blood (FPB), was validated based on international guidelines. Selectivity, carryover, and within/between-run accuracy and precision were in accordance with the recommendations. The matrix effect was evaluated at three different hematocrit levels (HT: 20%, 40%, 60%) and the coefficients of variation did not exceed 15%. Dilution integrity (1:10 and 1:20) was given for all analytes except lisinopril, yet for lisinopril, the therapeutic range was already covered by the calibration range. Long-term stability in VAMS tips was tested for 2 weeks at 24 °C in the dark and revealed no degradation of analytes. The proof of concept was performed by analyzing 35 intakes of ACE-inhibitors and diuretics in 18 VAMS and matched plasma samples. Hereby, determined concentration in FPB and plasma cannot be used interchangeably, and thus specific reference ranges for whole blood must be established. Nevertheless, the VAMS-based strategy was shown to be suitable for assessing adherence of all classes of antihypertensive drugs used in the guidelines to manage hypertension.

**Graphical abstract:**

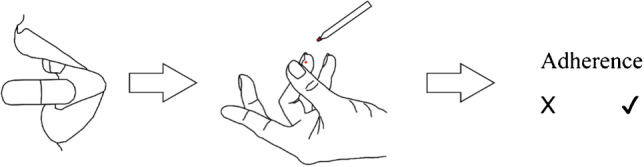

**Supplementary Information:**

The online version contains supplementary material available at 10.1007/s00216-022-04394-9.

## Introduction

Hypertension is the most prevalent, modifiable risk factor of cardiovascular disease, accounting for over a million deaths globally in 2019 [1, 2]. However, non-adherence to antihypertensive drugs (AHD) can be found in up to 50% of the treated patients and associates with uncontrolled blood pressure, poor clinical outcomes, and an increase in disease burden and costs to the healthcare system [3–5]. Consequently, the current guidelines for the management of arterial hypertension recommend interventions to improve adherence in all patients [6]. Physicians’ perception on patient’s adherence has been shown to be insufficient and often leads to an overestimation of adherence [7]. Therefore, adherence assessment is recommended in resistant hypertension and in patients with inadequate response after addition of an AHD. Commonly used indirect methods to assess adherence such as self-reporting or pill counting systems are known to be inaccurate, whereas direct measurements of drug concentrations in the patient’s blood are objective and can provide quantitative information [8, 9]. A study by Gupta et al. demonstrated that liquid chromatography-mass spectrometry (LC-MS)-based analyses of urine or blood of non-adherent patients improve their adherence with consecutive meaningful reductions in blood pressure [10]. Hereby, assessing adherence based on blood concentrations with individually calculated lower dose-related concentrations (DRC) is more accurate than using qualitative analysis alone [11, 12]. Up to date, several LC-MS methods for the quantification of AHD exist [13–16] but they are based on blood, plasma, or serum needing venipuncture, which can cause discomfort and anxiety and requires healthcare professionals. With dried blood spots (DBS), an alternative to venipuncture is known since 1963 [17]. Hereby, small drops of e.g., finger prick blood (FPB) can be obtained with a lancet by the patients themselves. Those drops have usually an unknown volume and are soaked on filter paper, dried, and can then be transferred even by mail to a laboratory [18, 19]. Another alternative to venipuncture is volumetric absorptive microsampling (VAMS), which is expected to maintain benefits of DBS like minimal invasion, cost effectiveness, and suitability for at-home sampling, but should overcome the volume inaccuracy and hematocrit (HT)-related effects of DBS [20–22]. VAMS devices consist of a plastic sample handler with a porous hydrophilic tip which can absorb different defined volumes of e.g., FPB (see electronic supplementary material, ESM, Fig. S1). VAMS were already used for e.g., screening procedures in different matrices, metabolic studies, therapeutic drug monitoring, and adherence monitoring [23–27].

This study aimed to close an analytical gap by improving a VAMS-based strategy [27] to allow assessing adherence to all classes of AHD listed in the current guidelines for hypertension treatment which include thiazide diuretics, calcium-channel blockers (CCBs), angiotensin-converting-enzyme (ACE) inhibitors, or angiotensin II receptor blockers (ARBs), and spironolactone in case of resistant hypertension [6, 28, 29]. The extraction of the VAMS tip and the analytical setup should be conserved but chromatographic conditions and mass spectrometry settings must be adapted and a proof of concept study had to be provided, including the comparison of FPB concentrations to matching plasma samples.

## Experimental

### Chemicals and other materials

The internal standards (IS) canrenone-d_4_, hydrochlorothiazide (HCT)-^13^C_6_, lisinopril-d_5_, torasemide-d_6_, and ramiprilat-d_5_ were purchased from Alsachim (Illkirch Graffenstaden, France), canrenone from European Pharmacopoeia (Strasbourg, France), HCT, furosemide, and torasemide from LGC (Luckenwalde, Germany), lisinopril from Merck (Darmstadt, Germany), ramiprilat from Aventis Pharma (Strasbourg, France), and enalaprilat from Sigma Aldrich (St. Louis, USA). All other chemicals (LC-MS grade or analytical grade) were from VWR (Darmstadt, Germany). Mitra VAMS with a 10 μL absorbing tip were purchased from Neoteryx (Torrance, USA). Blank blood, stabilized with ethylenediaminetetraacetic acid (EDTA), used for development and validation of the procedure was submitted to the authors’ laboratory for regular toxicological analysis and handled in accordance with the institutional protocol and regulations concerning data privacy and sample handling. FPB loaded VAMS, matching EDTA-stabilized blood samples, and medication plans for applicability studies were collected from volunteers with hypertension as part of their regular consultation at Saarland University Hospital, Homburg, Germany. All volunteers provided written informed consent and the local ethic committees approved the study (Homburg Hypertonie Register, 142/10).

### Calibrators, quality controls, internal standards, and preparation of VAMS and plasma

Stock solutions of each compound were prepared in duplicates at a concentration of 1 mg mL^−1^ in methanol, once for quality control (QC) and once for calibrator stock solution. The IS solution contained 200 ng mL^−1^ canrenone-d_4_, and 80 ng mL^−1^ HCT-^13^C_6_, lisinopril-d_5_, torasemide-d_6_, and ramiprilat-d_5_, each, in methanol. All solutions were stored in amber glass vials at −20 °C. The calibrator and QC working solutions were prepared by spiking the stock solutions in methanol and the final concentration in whole blood used to load VAMS tips are shown in Table 1. The volume of spiked solution did not exceed 5% of the total matrix volume [30]. To prepare calibrator and QC samples, 10 μL working solution were added to 190 μL blank human whole EDTA-stabilized blood or plasma and incubated for 30 min at 37 °C and 1500 rpm using a Thermoshaker Pro (CellMedia, Elsteraue, Germany) to allow plasma-protein binding and/or diffusion into red blood cells [27]. VAMS tips were loaded by holding them onto the surface of whole blood until completely soaked with an additional waiting time of 2 s [21]. VAMS tips were dried for at least 3 h at room temperature (24 °C) before sample preparation [31]. Different target HT values (20%, 40%, and 60%) in whole blood were prepared by adding or removing plasma after centrifugation for 11 min at 9660 × *g* [32, 33].

**Table 1:** Therapeutic reference ranges (therap. ref. range, ng/mL) in plasma and expected through concentrations (exp. ↓ conc.) (ng/mL) in serum at 24 h after the last antihypertensive drug intake. Final concentrations (ng/mL) of analytes in whole blood used to load calibrator (Cal) and quality control (QC) VAMS tips as well as weighting factors used for linear regression and the corresponding internal standard (IS) for calculation. (HCT, hydrochlorothiazide; LLOQ, lower limit of quantification; ULOQ, upper limit of quantification)

Analyte	Therap. ref range ng/mL [34]	Exp. ↓ conc. ng/mL	Cal 1	Cal 2	Cal 3	Cal 4	Cal 5	Cal 6	Cal 7	Cal 8 ULOQ	LLOQ	QC low	QC medium	QC high	Weighting	IS
Canrenone	50-250(-500)	6	30	40	50	70	100	150	200	250	30	90	125	225	Equal	Canrenone-d_4_
Enalaprilat	10-50(-100)	3	3	5	10	20	40	60	80	100	3	9	50	100	1/*x*^2^	Ramiprilat-d_5_
Furosemide	2-5(-10)	< 1	3	5	10	20	40	60	80	100	5	15	50	100	1/*x*^2^	Torasemide-d_6_
HCT	3-15	4	3	5	10	20	40	60	80	100	10	30	50	100	Equal	HCT-^13^C_6_
Lisinopril	(5-)20-70	3	3	5	10	20	40	60	80	100	5	15	50	100	1/*x*	Lisinopril-d_5_
Ramiprilat	1-40	2	3	5	10	20	40	60	80	100	10	30	50	100	1/*x*	Ramiprilat-d_5_
Torasemide	64-520(-2000)	3	3	5	10	20	40	60	80	100	3	9	50	100	1/*x*^2^	Torasemide-d_6_

### Sample preparation — VAMS

Sample preparation was performed according to a previous study [27]. Briefly, dried VAMS tips were stripped into amber 2 mL reaction tubes and 90 μL purified water followed by 10 μL IS solution were added. Samples were shaken for 15 min at 37 °C and 1500 rpm using a Thermoshaker Pro (CellMedia, Elsteraue, Germany) before 200 μL of precipitation agent (methanol:acetonitrile, 30:70, *v/v*) was added. Samples were shaken for 30 min at 1500 rpm and room temperature (24 °C) followed by 15 min of centrifugation at 15,000 × *g* and at freezing temperature of −10 °C. Finally, the supernatant was transferred into amber LC vials and a volume of 10 μL was injected onto the liquid chromatography-high-resolution tandem mass spectrometry (LC-HRMS/MS) system to be analyzed as described in “Instruments and settings”.

### Sample preparation — plasma

Sample preparation was performed according to a previous study [27]. Briefly, 10 μL plasma were transferred into an amber 2 mL reaction tubes and 90 μL purified water and 10 μL IS solution were added. Samples were shaken for 15 min at 37 °C and 1500 rpm using a Thermoshaker Pro (CellMedia, Elsteraue, Germany) before 190 μL of precipitation agent (methanol:acetonitrile, 30:70, *v/v*) was added. Samples were shaken for 30 min at 1500 rpm and room temperature (24 °C) followed by 15 min of centrifugation at 15,000 × *g* and at freezing temperature of −10 °C. Finally, the supernatant was transferred into amber LC vials and a volume of 10 μL was injected onto the LC-HRMS/MS system to be analyzed as described in “Instruments and settings”.

### Instruments and settings

All samples were analyzed using a Thermo Fisher Scientific (TF, Dreieich, Germany) Dionex UltiMate 3000 consisting of a degasser, a quaternary pump, a DL W2 wash system, and an HTC PAL autosampler (CTC Analytics AG, Zwinger, Switzerland), coupled to a TF Q-Exactive system equipped with a heated electrospray ionization (HESI)-II source. Mass calibration was done prior to analysis according to the manufacturer’s recommendations using external mass calibration. Gradient elution was performed on a TF Accucore Phenyl-Hexyl column (100 mm × 2.1 mm, 2.6 μm particle size). The mobile phase A consisted of 2 mM aqueous ammonium formate containing formic acid (0.1%, *v/v*, pH 3) and mobile phase B consisted of 2 mM aqueous ammonium formate with acetonitrile:methanol (50:50, *v/v*) plus formic acid (0.1%, *v/v*), and water (1%, *v/v*). The gradient was set as follows: 0–0.4 min hold 99% A, 0.4–0.7 min from 99% A to 83% A, 0.7–4.5 min from 83% A to 55% A, 4.5–7.3 min 55% A to 1% A, 7.3–8.5 min hold 1% A, and 8.5–9.4 hold 99% A. The flow rate was set as follows: 0–7.3 min 0.6 mL min^−1^, 7.3–8.5 min from 0.6 to 0.8 mL min^−1^, and 8.5–9.4 min 0.6 mL min^−1^. Chromatography was performed at 40 °C. The HESI-II source conditions were as follows: ionization mode, positive or negative; sheath gas flow rate, 60 arbitrary units (AU); auxiliary gas flow rate, 10 AU; spray voltage 4.00 kV; auxiliary gas heater temperature, 320 °C; ion transfer capillary temperature, 320 °C; and S-lens RF level 60.0.

Mass spectrometry analysis was performed using parallel reaction monitoring (PRM) in positive and negative mode with an inclusion list containing masses of interest. The settings for PRM data acquisition were as follows: resolution, 17,500; automatic gain control (AGC) target 2e^5^; maximum injection time (IT) 250 ms; isolation window, 2.0 *m/z*; and high-energy collisional dissociation (HCD) with normalized collision energy (NCE), 30 e.V. TF Xcalibur Qual Browser software version 2.2 was used for data evaluation. Precursor ion masses (*m/z*) used for the inclusion list and time windows for the detection are represented in the ESM in Table S1.

### Method validation

The method was validated according to international recommendations, including the European Medicines Agency (EMA) guideline on Bioanalytical Method Validation and the International Association of Therapeutic Drug Monitoring and Clinical Toxicology (IATDMCT) guideline for Development and Validation of Dried Blood Spot-Based Methods for Therapeutic Drug Monitoring [30, 35]. TF Xcalibur Quan browser version 2.2 and Microsoft Excel version 16 (Microsoft, Redmond, USA) were used to perform the statistical evaluation.

Ion suppression or enhancement due to coeluting analytes or coelution with IS was tested at a concentration of 10 ng mL^−1^ each in methanol. Peak areas of each analyte and each IS were determined (*n* = 3) once in absence and once in presence of the coeluting substance. Hereby, coeluting should neither enhance nor suppress peak areas more than 15% [36].

To test for selectivity of the method, blank FPB VAMS samples from six drug-free individuals were analyzed. Processed samples were analyzed for possible false-positive results or peak interferences with analytes or IS. Carryover was tested by injecting two blank matrix samples after analysis of the highest calibrator (*n* = 3) and after a sample having a concentration 10-times higher than the highest calibrator (*n* = 3). Interfering signals of analytes in blank matrix for selectivity and carryover testing should be < 20% of the lower limit of quantification (LLOQ) and < 5% of the IS [35].

Calibration ranges are represented in Table 1. Calibrators were prepared by spiking blank matrix with eight individual calibrator solutions. The sample preparation was performed as described in “Sample preparation — VAMS” or “Sample preparation — plasma”. Different weighting factors (equal, 1/*x*, or 1/*x*^2^) were tested by back calculation of calibrators and fitting of QCs. If the calibration standard does not meet the criteria, it should be rejected for regression analysis. However, at least 75% of the calibration standards must fulfill these criteria [35].

Accuracy and precision of the analytical method were determined for the LLOQ, QC low, QC medium, and QC high levels. Separately prepared stock solutions were used to spike QC samples and calibrators independently. QC concentrations were back-calculated via calibration curves and compared to nominal values. Within-run accuracy and precision were performed by analyzing five sample replicates of each level, obtained within a single run and between-run accuracy and precision were performed by analyzing five sample replicates of each level on three runs analyzed on three different days. Determined mean concentrations of QC levels should be within ± 15% of the nominal values (± 20% for LLOQ) for positive assessment of accuracy and for precision, the coefficient of variation (CV) should be within 15% (20% for LLOQ). CV for between-run precision was calculated by dividing the standard deviation of the mean concentrations of each day (3 days with *n* = 5) by the determined overall mean concentration.

Dilution integrity of the final extract was determined by spiking blank whole blood samples (*n* = 5) with a concentration 10-times higher than the highest calibrator. Extracts were diluted 1:10 and 1:20 with extracted zero sample (containing IS but no analytes). Accuracy and precision were determined.

The matrix effect (ME) and the recovery (RE) were investigated using blood samples (*n* = 6 in total) at low and high QC levels. EDTA-stabilized whole blood with HT 40% (*n* = 6), HT 20% (*n* = 3), and HT 60% (*n* = 3) was used to load the VAMS tips. The ME and RE were determined according to Matuszewski et al. [37]. The ME was calculated by the ratio of the peak area in the presence of matrix (blank matrix spiked after extraction) to the peak area in absence of matrix (pure analyte solution). The RE was determined by calculating the ratio of the peak area extracted with the matrix (blank matrix spiked before extraction) to the peak area in the presence of matrix (blank matrix spiked after extraction) [37]. The CV of the ME and the RE should be within 15% [35].

Stability of stock solutions in methanol at −20 °C was tested for all analytes over a time of 8 weeks (*n* = 3). Furthermore, the long-term stability (2 weeks, 24 °C, in a dark box) in the VAMS tips and the autosampler stability (48 h, 10 °C) of processed samples were investigated (*n* = 3) using the low and high QC levels. Determined QC concentrations should be within ± 15% of the nominal concentration when analyzed immediately after preparation and after evaluated storage conditions using a freshly prepared calibration curve.

### Proof of concept

As a proof of concept of the methods applicability, authentic VAMS tips soaked with FPB and matching plasma samples of 18 individuals were analyzed. EDTA-stabilized blood was centrifuged for 5 min at 1645 × *g* to generate plasma. For the assessment of adherence, cut-off concentrations were determined using DRC-factors calculated for the corresponding dosing interval of medication [12, 38]. Since most subjects took their medication in the morning and samples were taken about noon, the DRC-factor for *∆t* = 6h (*∆t* = interval between drug intake and blood sampling) was calculated and used for the calculation of cut-off concentrations for the adherence assessment (see ESM equation S1 and Table S4).

## Results and discussion

Urine can be obtained non-invasively and is often described as the gold standard to detect non-adherence due to a longer time window available for drug detection compared to blood [39–41]. However, methods for urine analysis are mostly qualitative in nature and may lead to overestimation of adherence due to excretion of drugs exceeding the dosing interval manyfold [38]. The assessment of adherence based on blood, serum, or plasma drug concentrations is expected to be more accurate, particularly if a DRC approach is used [12, 38, 42]. Therefore, this study aimed to close an analytical gap to allow assessing adherence of the most frequently prescribed classes of AHD using just one VAMS tip [6, 27–29]. Parent compounds or active metabolites were used for quantification. In contrast to therapeutic drug monitoring, the quantification of the parent compound or the active metabolites only is sufficient to assess adherence correctly.

### Method validation

Chromatographic separation within 7.5 min using a total run time of 9.4 min is represented in Fig. S2 (ESM). To identify the analytes, retention times and full MS^2^ spectra were considered. MS^2^ spectra were visually compared with a database considering the presence of the most abundant fragment ions and their relative abundance [43]. Full MS^2^ spectra instead of only ion ratios were used for identification to increase selectivity. For quantification, peak area ratios of specific fragment ion in MS^2^ of the analyte and the IS were used (ESM Table S1). Each analytical batch consisted of a zero sample, containing IS but no AHD, eight calibration standards, three QC levels, and the patient samples.

Complete chromatographic separation could not be achieved without changing the analytical setup. Therefore, possible ion suppression or enhancement of coeluting analytes and their corresponding IS, as well as the coelution of torasemide and ramiprilat, were investigated. Thereby, only lisinopril (+20%) and lisinopril-d_5_ (+38%) showed ion enhancement. However, the IS lisinopril-d_5_ was only used for the quantification of lisinopril, thus effects in unknown samples and calibration samples are expected to be similar and therefore compensated. No other analyte or IS revealed ion suppression or enhancement exceeding ± 15% (−11% to +11%) of peak areas [36]. We had no access to corresponding IS for enalaprilat and furosemide; therefore, the IS ramiprilat-d_5_ was used for the quantification of enalaprilat and torasemide-d_5_ was used for the quantification of furosemide.

Since no interfering signals from endogenous compounds or false-positive results could be detected using the retention time in combination with a library spectrum as reference, the selectivity of the method was given [43]. No carryover was observed after injection of the highest calibrator or after injection of a 10-times higher concentration. However, samples following an injection of an even higher concentration should be reanalyzed after a washing run with extracted blank matrix.

After testing different weighting factors, a linear (weighted) calibration model could be used for all analytes (equal, 1/*x*, or 1/*x*^2^, see Table 1). Results for the within- and between-day accuracy and precision are represented in Table 2. All analytes met the criteria according to the EMA guideline of a mean concentration within ± 15% (± 20% for LLOQ) of the nominal concentration and the CV did not exceed 15% (20% for LLOQ) [35]. In case of furosemide, HCT, and ramiprilat, the lower therapeutic range could not be completely covered by the method. For canrenone and lisinopril, the therapeutic range is covered; however, the expected trough concentrations cannot be achieved (see Table 1).

**Table 2:** Within- and between-day accuracy and precision of the lower limit of quantification (LLOQ), quality control (QC) low, QC medium (mid), and QC high (*n* = 5 at three different days). Dilution integrity with processed blank matrix containing IS (zero sample). 1:10 dilution and 1:20 dilution of a 10-times higher concentration than the upper limit of quantification (*n* = 5). (CV, coefficients of variation; HCT, hydrochlorothiazide)

Analyte		Relative mean concentration (accuracy), %; CV (precision), %								
	Within-day				Between-day				Dilution	
	LLOQ	QC low	QC mid	QC high	LLOQ	QC low	QC mid	QC high	1:10	1:20
Canrenone	81; 17	87; 15	91; 15	88; 9	86; 11	87; 1	91; 1	89; 6	106; 5	86; 8
Enalaprilat	115; 13	93; 13	93; 15	93; 10	101; 12	98; 4	97; 4	97; 10	102; 8	108; 10
Furosemide	81; 10	86; 12	87; 7	92; 10	95; 14	94; 8	92; 5	94; 7	86; 8	92; 7
HCT	81; 18	100; 15	112; 7	104; 12	93; 17	100; 0	107; 8	101; 8	99; 10	90; 12
Lisinopril	108; 9	85; 10	98; 11	104; 9	112; 6	93; 8	105; 8	106; 9	118; 4	131; 6
Ramiprilat	90; 4	100; 8	106; 13	106; 8	94; 4	101; 2	111; 4	109; 9	89; 5	99; 10
Torasemide	99; 7	91; 8	91; 9	93; 11	94; 11	95; 5	101; 9	97; 9	91; 7	93; 8

Dilution integrity was tested by dilution (1:10 and 1:20) of a processed sample, having a concentration ten-times higher than the ULOQ, with processed zero sample (blank matrix containing IS). Only lisinopril exceeded recommended criteria for accuracy with 18% for the dilution factor of 1:10 and 31% for the dilution factor of 1:20. However, the therapeutic range for lisinopril is already covered by the calibration. All other analytes met recommended criteria for accuracy and precision [35]. The advantage of the zero sample compared to the precipitation agent spiked with IS for the dilution of the final extract was shown in previous studies [26, 27]. Including the dilution factor, the upper part of the therapeutic range of canrenone and torasemide can be covered. For the assessment of adherence, this may not be mandatory since a patient can be considered as adherend if the determined concentration of the drug is above the expected trough concentration, an exact quantification is not essential.

Determined ME and RE values, as well as corresponding CVs for the analytes, are represented in Table 3, and those of the IS are represented in ESM Table S2. At different HT values, ME of analytes varied between 71% for canrenone at HT 40% and 226% for lisinopril at HT 20%. However, these effects were found to be reproducible for all analytes at all three tested HT values with CVs within 15%. Thus ME for normal HT fluctuations between 36 and 53% is reproducible [44]. ME of the IS was also reproducible with CVs within 15%. However, nominal values of ME for most analytes vary largely between QC levels and different HT values. Of note, the nominal value of the ME is higher for ACE-inhibitors which have acidic groups in their chemical structure. The RE was found to be reproducible for all analytes at the three investigated HT values with CVs within 15% except for enalaprilat at QC low and HT 40% with a CV of 16%. Nominal values of RE were comparable between QC levels and different HT levels for most analytes. Hereby, nominal values at QC high level at HT 60% were inferior for all analytes. Reproducibility of ME and RE seems to be sufficient for the purpose of adherence assessment.

**Table 3:** Matrix effect, recovery, and coefficients of variation (CV) of the analytes of low- and high-quality controls (QC) for VAMS at different hematocrit (HT) values (*n* = 6 at HT 40%; *n* = 3 at HT 20% and HT 60%). (HCT, hydrochlorothiazide)

Analyte	Matrix effect, %; CV, %						Recovery, %; CV, %					
	QC low			QC high			QC low			QC high		
	HT20%	HT40%	HT60%	HT20%	HT40%	HT60%	HT20%	HT40%	HT60%	HT20%	HT40%	HT60%
Canrenone	79; 7	79; 15	78; 11	74; 6	71; 7	73; 1	75; 9	79; 13	88; 11	92; 8	85; 11	68; 14
Enalaprilat	142; 14	159; 12	168; 5	122; 9	118; 7	124; 8	84; 14	94; 16	94; 13	101; 10	95; 15	80; 9
Furosemide	107; 7	123; 11	133; 15	114; 3	105; 8	111; 7	110; 13	91; 12	108; 14	95; 9	94; 13	86; 11
HCT	128; 4	128; 13	119; 13	109; 11	106; 7	113; 4	87; 8	98; 12	95; 14	110; 9	97; 12	82; 13
Lisinopril	226; 12	201; 13	178, 13	169; 8	163; 10	176; 7	86; 8	97; 9	94; 14	97; 6	92; 12	71; 9
Ramiprilat	137; 15	135; 5	135; 11	121; 4	119; 5	126; 3	79; 13	87; 15	84; 7	98; 9	89; 12	73; 7
Torasemide	105; 14	134; 11	108; 5	96; 2	112; 11	111; 4	99; 14	90; 12	102; 11	99; 9	90; 14	77; 10

Stability of analytes in stock solutions was tested and all analytes were found to be stable over at least 8 weeks at −20 °C in amber glass vials. Results of short- and long-term stability are given in ESM Table S3. No analyte showed a degradation over 15% of the nominal concentration for the low and high QC levels after storage for at least 48 h of processed samples in the autosampler at 10 °C. This is of advantage if long-lasting analytical series are planned; however, the duration of a batch should not exceed this period since autosampler stability can only be guaranteed for 48 h based on these experiments. Furthermore, all analytes showed good long-term stability in the sampling device. After 2-weeks storage at 24 °C in a dark box, no analyte showed a degradation over 15% of the nominal concentration. However, spiked samples may not always display the same stability profile as actual samples [30].

In summary, the present method allowed a quantification of enalaprilat and torasemide over their complete therapeutic range and at the expected trough concentrations (see Table 1) in whole blood after VAMS sampling. For canrenone and lisinopril, the therapeutic range was covered; however, the expected trough concentration cannot be met. For furosemide, HCT, and ramiprilat, only parts of the therapeutic range were covered. The references of therapeutic ranges were those for plasma and the calculated expected through concentration were calculated for serum since reference ranges for whole blood, which is the case for FPB, were not available. In most cases, serum and plasma analyte concentrations are the same [45]; however, considering the different nature of matrices is of importance. Presumably, reference ranges for VAMS are the same as for DBS, since in both cases, FPB is typically used as sample matrix.

### Proof of concept

As a proof of concept, 18 authentic matching VAMS tips soaked with FPB and plasma samples were analyzed. Hereby, monitoring of 35 intakes of seven different ACE-inhibitors and diuretics was carried out (enalapril *n* = 2, furosemide *n* = 3, HCT *n* = 3, lisinopril *n* = 1, ramipril *n* = 10, spironolactone *n* = 5, torasemide *n* = 11).

The method was only validated for VAMS as sample matrix and not for plasma. Sample preparation was the same as for the VAMS sample, and therefore, only the matrix was different (dried whole blood vs. plasma). During extraction, the VAMS tip was exchanged by 10 μL of plasma and 190 μL precipitation agent instead of 200 μL were used to compensate the volume. Thus, to get an impression of concentration differences between dried whole blood and plasma, validation of plasma as sample matrix would not be justified regarding effort and expenses.

Table 4 shows the results of adherence assessment of ACE-inhibitors and diuretics in FPB in comparison to plasma. DRC-factor for *∆t* = 6 h was calculated and used for the calculation of cut-off concentrations for the adherence assessment (see ESM equation S1 and Table S4). However, in individual cases, *∆t* was shorter than 6 h. Adherence was assessed as positive if determined concentrations were above the calculated DRC. Adherence cannot be assessed if the calculated DRC and determined concentrations are below the LLOQ. This study used the lower DRC-factor for calculation of cut-off concentrations. Hereby, interindividual variability in drug elimination are considered by incorporating the standard deviation (SD) of the total clearance into equations (see ESM equation S1). A limitation of this method is that it is a theoretical calculation based on pharmacokinetic properties of different drugs. Therefore, cut-off concentrations should be clinically validated against measured drug concentrations in patients known to be adherend since pharmacokinetics are influenced by e.g., age, comorbidity, and renal clearance [12]. Additionally, different properties of whole blood and plasma are not considered in this equation.

**Table 4:** Quantification of antihypertensive drugs (ng mL^−*1*^) using finger prick blood sampled by VAMS and plasma and assessment of adherence using cut-off concentrations calculated with use of dose-related concentration (DRC) factor. Prescribed medication and mode of intake provided by medication plans. For the intake of enalapril, ramipril and spironolactone concentrations were determined for the active metabolites enalaprilat, ramiprilat, and canrenone, respectively. (conc., concentration; HT, hematocrit; HCT, hydrochlorothiazide; ↓, classified as nonadherent; -, no data; i.v., intravenous)

Patient	HT, %	Medication	Mode of intake	Conc. in VAMS, ng mL^−1^	Conc. in plasma, ng mL^−1^	DCR-factor	Cut-off conc. using DRC, ng mL^−1^
1	25	Ramipril 2.5 mg	2-0-0	< 10	< 10	1.6	8
2	38	Ramipril 2.5 mg	1-0-1	< 10 ↓	< 10 ↓	2.5	13
3	26	Torasemide 5 mg Ramipril 5 mg	1-0-0 1-0-0	51 ↓ 15 ↓	76 ↓ 18 ↓	18.5 1.6	93 25
4	32	Torasemide 10 mg Ramipril 2.5 mg	1-0-0 1-0-0	120* ↓ < 10	*140* ↓ < 10	18.5 1.6	185 4
5	38	Enalapril 10 mg	1-0-1	12 ↓	19 ↓	3.0	60
6	36	Torasemide 10 mg Ramipril 10 mg	1-0-0 1-0-0	480* 11 ↓	610* 15 ↓	18.5 1.6	185 16
7	36	Torasemide 10 mg Lisinopril 20 mg	1-0-0 1-0-1	390* > 100	200* > 100	18.5 2.9	185 114
8	46	Torasemide 10 mg Spironolactone 25 mg	1-0-0 1-0-0	280* < 30	200* < 30	18.5 0.5	185 13
9	42	Ramipril 5 mg HCT 12.5 mg	1-0-0 1-0-0	< 10 64	< 10 30	1.6 1.7	8 21
10	32	HCT 12.5 mg Ramipril 2.5 mg	1-0-0 1-0-0	67 < 10	34 < 10	1.7 1.6	21 4
11	48	Torasemide 10 mg Spironolactone 25 mg Ramipril 2.5 mg Furosemide i.v.	1-0-0 1-0-0 ½-0-0 -	250* < 30 < 10 < 5	380* 40 < 10 < 5	18.5 0.5 1.6 -	185 13 2 -
12	42	Torasemide 10 mg Ramipril 5 mg HCT 12.5 mg	½-0-0 1-0-0 1-0-0	200* < 10 89	160* < 10 23	18.5 1.6 1.7	93 8 21
13	44	Enalapril 2.5 mg Furosemide i.v.	1-0-1 -	6 ↓ < 5	7 ↓ < 5	3.0 -	8 -
14	38	Torasemide 10 mg Spironolactone 25 mg Ramipril 5 mg	1-0-0 1-0-0 1-0-0	140* ↓ < 30 < 10	140* ↓ < 30 10	18.5 0.5 1.6	185 13 8
15	40	Spironolactone 25 mg	1-0-0	< 30	< 30	0.5	13
16	40	Torasemide 10 mg Spironolactone 50 mg	1-½-0 1-0-0	270* 38	250* 33	- 0.5	- 25
17	28	Torasemide 10 mg Torasemide perfusion	1-0-0 -	> 2000*	> 2000*	18.5 -	-
18	44	Furosemide i.v Torasemide 10 mg	- 2-1-0	100 120*	130* 58	- -	- -

*Conc. determined after dilution

Mode of intake described as number of tablets taken in the morning-noon-evening

Determined concentration of ACE-inhibitors and diuretics differed between VAMS and plasma. Enalaprilat (*n* = 2), furosemide (*n* = 1), and ramiprilat (*n* = 3) concentrations were determined higher in plasma as sample matrix compared to VAMS. For HCT (*n* = 3), the determined concentration in VAMS exceeded those determined in plasma. For canrenone, the determined concentration was once higher and once lower in VAMS compared to plasma. Neither for torasemide a trend could be seen. Once the determined concentration was equal in both matrices, in five cases, the determined concentration in VAMS was below plasma concentrations, and five times the determined concentration in VAMS exceeded that of plasma. For lisinopril (*n* = 1), concentration in VAMS and plasma exceeded calibration. For all other intakes, determined concentrations were below the LLOQ for both matrices. For patient 7, lisinopril exceeded the therapeutic range and the calibration range. This can be due to a short time between medication intake and sampling (*∆t*). For patient 17, torasemide exceeded calibration range even after dilution (1:20); however, this was most likely caused by the additional torasemide perfusion. However, no differences in adherence assessment between matrixes could be observed.

FPB consists of whole blood were total drug levels are measured, whereas plasma measurements do not include intracellular drug levels [46]. Since some drugs can largely distribute into red blood cells, considering the different matrices is important [47]. Yet, specific reference ranges are more important in the context of therapeutic drug monitoring as in the context of adherence monitoring. Peeters et al. showed that a fixed correction factor between whole blood and plasma was not possible for all AHD and Jacobs et al. also described differences in determined concentrations of AHD in FPB sampled by VAMS and matching plasma samples [27, 48]. In conclusion, quantification results in FPB and plasma cannot be used interchangeably and many samples, from patients known to be adherend, sampled at trough concentration will be needed to establish reference ranges for the assessment of adherence. Nevertheless, the current results demonstrate that VAMS are suitable tool for adherence monitoring of ACE-inhibitors and diuretics, and may replace venipuncture in the future.

Six patients were classified as non- or partially adherent using DRC-factors for calculation of cut-off concentrations by using FPB sampled by VAMS. Classification of adherence was the same using plasma as sample matrix. Compared to other studies, the high rate of adherence/partial adherence is due to the sampling of inpatients, where medication intake is usually supervised [39, 49, 50]. The six patients classified as non- or partially adherend show the limitation of using the theoretical calculation of expected concentration as a cut-off concentration. Hereby, experimental values determined in a clinical validation should be taken into consideration to determine cut-off concentrations. Furosemide and in one case torasemide were applied intravenously (i.v.) and the expected trough concentrations can therefore not be calculated by equation S1 (ESM). Determined concentration can therefore not be used to assess adherence; however, in case of i.v. applications, adherence is anyway not questionable. Furthermore, for patients 16 and 18, torasemide cut-off concentrations cannot be calculated due to an unequal dosing interval and changing dosage over the day. This showed that theoretical calculations always have their limits.

## Conclusions

Adherence monitoring is of great interest within healthcare and assessing adherence objectively is still a challenge and various methods for adherence assessment exist, all with their specific advantages and disadvantages [51]. An analytical gap in adherence assessment of AHD could successfully be closed, with some limitations. Proof of concept was conducted by monitoring 35 intakes of seven different AHD in VAMS tips and matched plasma samples of 18 patients. In addition, all analytes showed a sufficient stability in dried matrix using VAMS as a sampling strategy. However, concentration in FPB sampled by VAMS and plasma differ in most cases and specific reference ranges for whole blood must be established. The current method allows at-home sampling and thus acceptance by the patient. Furthermore, hospital staff has no longer to perform venous blood sampling. The method allows now to bioanalytically assess the adherence of all guideline-listed antihypertensive drugs using just a single VAMS. Additionally, the differences between determined concentrations in finger prick blood and plasma as sample matrix were reported forming the foundation for further investigations of specific reference ranges for a broad range of antihypertensive drugs in finger prick blood.

## Supplementary information

ESM 1 (PDF 2579 kb)
