# Supplementary material for: Closing the gap — development of an analytical methodology using volumetric absorptive microsampling of finger prick blood followed by LC-HRMS/MS for adherence monitoring of antihypertensive drugs
Source: Anal Bioanal Chem. 2022 Nov 1;415(1):167–77. doi: 10.1007/s00216-022-04394-9 (PMC9816235; doi:10.1007/s00216-022-04394-9)
Supplement: Supplementary file 1 — (PDF 2579 kb) [file 216_2022_4394_MOESM1_ESM.pdf]

Electronic supplementary material

Closing the gap – Development of an analytical methodology using volumetric absorptive microsampling of finger prick blood followed by LC-HRMS/MS for adherence monitoring of antihypertensive drugs

Cathy M. Jacobs, Michael Kunz, Felix Mahfoud, Lea Wagmann, Markus R. Meyer

Table S1: Calculated exact precursor ion masses used in the inclusion list for positive or negative ionization mode, accurate fragment ion mass used for quantification, retention times of the antihypertensive drugs and internal standards (IS), and time windows for compound detection. (HCT: hydrochlorothiazide; positive: pos; negative: neg)

**Analyte**	**Precursor ion mass, *m/z***	**Fragment ion mass, *m/z* for quantification**	**Retention time, min**	**Polarity**	**Time window, min**
Canrenone	341.2111	341.2111	7.25	pos	6.50-9.00
Canrenone-d_4 (IS)	345.2362	345.2362	7.24	pos	6.50-9.00
Enalaprilat	349.1758	206.1169	3.53	pos	0.50-4.20
Furosemide	328.9998	204.9827	5.50	neg	4.20-6.50
HCT	295.9567	295.9567	2.49	neg	0.50-4.20
HCT-$^{13}C_6$ (IS)	301.9772	301.9772	2.52	neg	0.50-4.20
Lisinopril	406.2336	84.0812	2.93	pos	0.50-4.20
Lisinopril-d_5 (IS)	411.2650	84.0812	2.95	pos	0.50-4.20
Ramiprilat	389.2071	206.1169	5.08	pos	4.20-6.50
Ramiprilat-d_5 (IS)	394.2385	211.1485	4.91	pos	4.20-6.50
Torasemide	349.1328	264.0806	5.05	pos	4.20-6.50
Torasemide-d_6 (IS)	355.1705	264.0806	5.02	pos	4.20-6.50

Table S2: Matrix effect, recovery, and coefficients of variation (CV) of internal standards for VAMS at different hematocrit (HT) values (n=6 at HT 40%; n=3 at HT 20% and HT 60%). (HCT: hydrochlorothiazide)

	Matrix effect, %; CV, %			Recovery, %; CV, %		
**Analyte**	**HT 20%**	**HT 40%**	**HT 60%**	**HT 20%**	**HT 40%**	**HT 60%**
Canrenone-d_4	61; 12	73; 11	63; 5	97; 5	97; 10	100; 2
HCT-$^{13}C_6$	95; 9	99; 11	102; 9	111; 13	110; 9	110; 8
Lisinopril-d_5	269; 14	219; 14	240; 11	95; 10	95; 8	96; 1
Ramiprilate-d_5	91; 10	102; 11	111; 12	109; 6	95; 6	96; 2
Torasemide-d_6	96; 7	100; 6	92; 5	111; 4	99; 11	107; 12

Table S3: Autosampler stability for 48 h at 10°C (n=3) and two-week stability in the sampling device at 24°C in a dark box (n=3). (QC: quality control; CV: coefficient of variation; HCT: hydrochlorothiazide)

**Analyte**	**Relative mean concentration, %; CV, %**			
	**QC low**		**QC high**	
	**48 h 10°C**	**2 weeks 24°C**	**48 h 10°C**	**2 weeks 24°C**
Canrenone	94; 7	87; 4	94; 1	86; 3
Enalaprilat	99; 9	88; 7	104; 4	91; 6
Furosemide	90; 9	105; 6	92; 4	105; 7
HCT	96; 13	102; 13	96; 4	115; 4
Lisinopril	93; 11	86; 8	109; 1	115; 3
Ramiprilat	105; 5	103; 4	114; 4	114; 8
Torasemide	106; 10	95; 7	103; 5	94; 6

Table S4: Dose related concentration (DRC) factors for a dosing interval (di) of 12 h or 24 h and sampling time after intake (Δt) of 6 h or 24 h [1, 2].

**Drug**	**Canrenone**	**Enalaprilat**	**Furosemide**	**Hydrochlorothiazide**	**Lisinopril**	**Ramiprilat**	**Torasemide**
**Daily dose (mg) low**	25	5	20	12.5	5	2.5	5
**Dosing interval (h)**	12/24	12/24	12/24	12/24	12/24	12/24	12/24
**Bioavailability (%)**	25	41	71	71	25	48	79
**Clearance (mL min^{-1})**	301	141	116	343	106	203	33.97
**SD clearance (mL min^{-1})**	130	43	41	77	13	57	9.80
Clearance -1 SD low (ml min^{-1})	*171*	*98*	*75*	*266*	*93*	*146*	*24.17*
Clearance +1 SD (ml min^{-1})	*431*	*184*	*157*	*420*	*119*	*260*	*43.77*
Elimination constant	*0.000717544*	*0.00105022*	*0.008886502*	*0.001444057*	*0.000962704*	*0.000825175*	*0-003122285*

di=24 h, Δt=6 h

Minimal concentration (ng mL^{-1})	12.48	10.28	32.79	20.74	9.53	4.07	92.60
DRC factor ((ng mL^{-1}) mg^{-1})	0.50	2.06	1.64	1.66	1.91	1.63	18.52

di=12 h, Δt=6 h

Minimal concentration (ng mL^{-1})	19.92	15.11	32.85	28.07	14.30	6.32	102.38
DRC factor ((ng mL^{-1}) mg^{-1})	0.80	3.02	1.64	2.25	2.86	2.53	20.48

Expected trough concentrations
di=24 h, Δt=24 h

Minimal concentration (ng mL^{-1})	5.75	3.31	<1	4.36	3.37	1.67	3.18

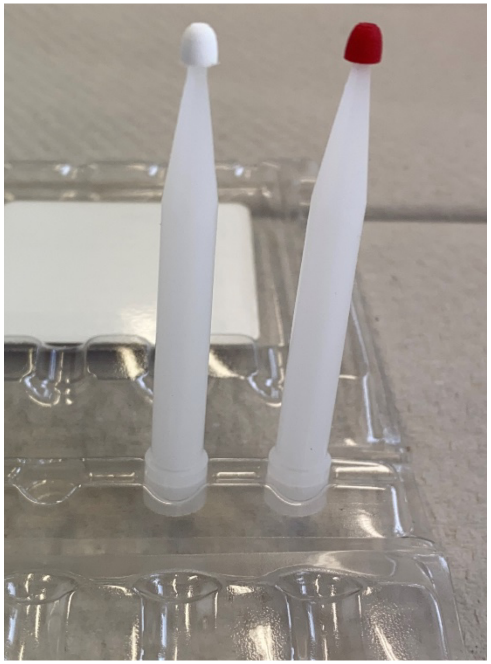

Figure S1: Volumetric absorptive microsampling (VAMS) devices before soaking blood (left) and fully soaked with whole blood (right)

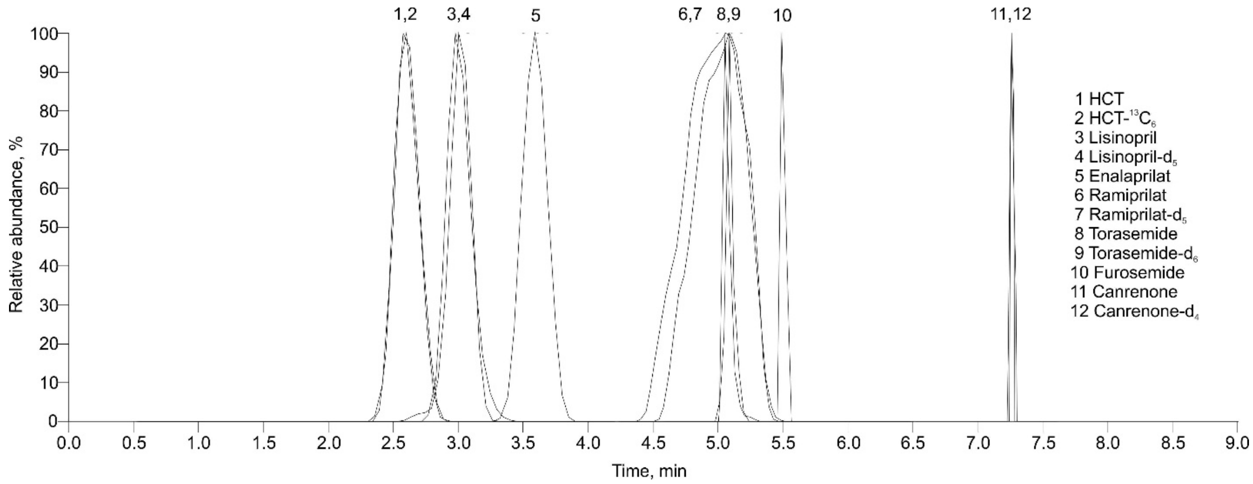

Figure S2: Chromatographic separation of all analytes at lower limit of quantification. All peaks at 100% relative abundance. (HCT: hydrochlorothiazide)

Equation S1: Calculation of dose related concentration (DRC) factor [1].

$$C_t = \left[\left(\frac{D}{di}\right) * \left(\frac{F}{Cl + SD}\right)\right] * \left[\frac{k_e * di}{1 - e^{-k_e * di}}\right] * \left(e^{-k_e * \Delta t}\right)$$

$$DRC\ factor = \frac{C_t}{D} = \left[\left(\frac{1}{di}\right) * \left(\frac{F}{Cl + SD}\right)\right] * \left[\frac{k_e * di}{1 - e^{-k_e * di}}\right] * \left(e^{-k_e * \Delta t}\right)$$

C_t: through concentration at 24 h
D: dose
di: dosing interval
k_e: elimination rate constant
Δt: interval between drug intake and blood sampling
F: bioavailability
Cl: total body clearance
SD: standard deviation

References

[1] S. Rognstad, C.L. Soraas, O.U. Bergland, A. Hoieggen, M. Strommen, A. Helland, M.S. Opdal, Establishing Serum Reference Ranges for Antihypertensive Drugs, Ther. Drug Monit., 43 (2021) 116-125.
[2] S. Ritscher, M. Hoyer, C. Wunder, N. Obermuller, S.W. Toennes, Evaluation of the dose-related concentration approach in therapeutic drug monitoring of diuretics and beta-blockers - drug classes with low adherence in antihypertensive therapy, Sci. Rep., 9 (2019) 15652.
